# Effect of ultrasonic activation on four different sealers for intra-tubular penetration to root dentin

**DOI:** 10.6026/9732063002001593

**Published:** 2024-11-30

**Authors:** Bhardwaj Nikhil, Jain K Rajnish, Khetarpal Ambica, Suri Shally, Arora Rachita, Singh Avishek

**Affiliations:** 1 Department of Conservative Dentistry and Endodontics, PDM Faculty of Dental Sciences and research institute, PDM University, Bahadurgarh, Haryana, India; 2 Department of Periodontology and Oral Implantology, Baba Jaswant Dental College, Hospital and Research Institute, Ludhiana, Punjab, India; 3 Department of Public Health Dentistry, NIMS Dental College and Hospital Jaipur, Rajasthan, India

**Keywords:** Sealer, ultrasonic, root canal, dentine penetration

## Abstract

The purpose of this study was to assess how endodontic sealers' ultrasonic activation (UA) affected dentin tubule penetration. The sample size came out to be 48 *i.e.* 12 samples in each group (four groups based on four sealers) which were further subdivided into 3 subgroups (4 samples in each subgroup). It was found that ultrasonic activation greatly enhances sealer penetration. Further research is warranted to compare the effect of ultrasonic activation (UA) of four different sealers on dentin tubule penetration.

## Background:

The goal of root canal therapy is to lessen the microbial burden in the canal system in order to stop or manage an inflammatory process in the periapical tissues. Thus, after the disinfection procedures, three-dimensional filling of the main canal and its ramifications is necessary, preventing bacterial migration and proliferation in the canals or periodontium. In order to create a fluid-tight or hermetic seal throughout the canal, including the apical foramen and the irregularities in the canal, endodontic sealers are employed during the root canal obturation process. According to Grossman, an ideal root canal sealer should provide the following: as excellent seal when set, dimensional stability, a slow setting time to provide proper adherence with canal walls, insolubility to tissue fluid and biocompatibility [1]. The different level of residual moisture in the root canal has been shown to alter the sealing properties of conventional and resin- based sealers. Thus, the quality of adhesion between the root canal dentine and sealers may also be affected by the moisture conditions of the root canals before filling procedures [2]. Root canal sealers can be classified according to their composition: zinc oxide- and eugenol-based sealers; sealers containing calcium hydroxide; epoxy resin-based sealers; glass ionomer sealers; methacrylate resin-based sealers; or silicon- and bioceramic-based sealers [3]. Calcium hydroxide-based sealers are thought to slow the growth of microbes in the root canal space. Similarly, resin-based sealers have been introduced in an attempt to achieve bonding of the root filling with the root dentine thereby forming a mono block to better seal the root canal space [4]. Sealapex (calcium-hydroxide based sealer) stimulates the deposition of the calcified structure, including apical sealing after root canal treatment [5]. New calcium-silicate based endodontic sealers have been developed based on their excellent biological properties and bioactive potential. These sealers promote high pH, allow Calcium ion release and present bond strength similar to AH Plus. However, high solubility is also reported for ready-to-use calcium silicate-based endodontic sealer. Bio-C sealer is a new root canal sealer containing calcium silicates, calcium aluminate, calcium oxide, zirconium oxide, iron oxide, silicon dioxide and dispersing agent in its composition. According to its manufacturer, this sealer has biocompatibility; bioactivity; and high pH, radiopacity and flow values [6]. Epoxy-resin based has been commonly used as gold standard endodontic sealers due to its high bond strength to dentine, adequate radiopaque, flow, dimensional stability, low solubility and high resistance [7]. AH plus is an epoxy-resin based sealer with good sealing properties [8]. Endo-methasone-a zinc-oxide eugenol-based sealer has been valuable therapeutic material for an optimum sealing of the root canal and remains a perfect option in the obturation of root canals [9]. In order to improve the quality of obturation, ultrasonic activation of sealers has been proposed showing promising results. The activation occurs through the use of specific ultrasonic tips connected to devices the produces high frequencies vibrations (25-30 kHz) [10]. The ultrasonic energy has been used to improve the flow of various materials within root canals. Ultrasonic activation (UA) of an endodontic sealer promotes greater penetration into the dentinal tubules and improves the sealer/dentine interface. The acoustic micro streaming energy transmitted improves the cleaning ability of irrigating solutions, diffusion of medicaments and the interfacial adaption of root canal sealers [11].

Therefore, it is of interest to describe the effect of Ultrasonic activation on four different sealers on intratubular penetration to root dentin.

## Materials & Methods:

## Study design and the setting:

Study was conducted in Department of Conservative Dentistry and Endodontics in PDM dental college, Bahadurgarh, Haryana. Study was conducted during the period of March 2022 to May 2023 with sample size 48(calculated using G power version 3.1.9.6 programmed. Based on 80% power of the study and 5% type1 error and effect size of 0.64, the sample size came out to be 48 *i.e.* 12 samples in each group (four groups based on four sealers) which were further subdivided into 3 subgroups each depending on type of activation (4 samples in each subgroup). All clinical procedures and data collection was done in the Department of Conservative Dentistry and Endodontics, PDM Dental College, Bahadurgarh, Haryana.

## Inclusion and exclusion criteria:

Freshly extracted human teeth, Teeth should be caries free; there should be complete absence of any pulpal calcification. Whereas, teeth with any type of resorption whether internal or external, teeth with cracks on the surface were excluded from the study.

## Study procedure:

Extracted single rooted maxillary and mandible teeth with straight canals and fully formed apices without calcification and no previous endodontic treatment were chosen. Next, teeth were kept in 0.9% saline solution until the following methodological steps. The teeth were decoronated with a diamond disc under constant irrigation and the root was standardized to 16mm length. All teeth were placed in elastomeric blocks. A size #15 K-file was introduced into the root canal until its tip was visible in the apical foramen. Working length must be 1mm short of apical foramen. Root canal preparation was performed with the help of Neoflex files under 20ml of 2.5% sodium hypochlorite (NAOCL) irrigation followed by 5ml of ethylenediaminetetraacetic acid (EDTA) 17% during 5 minutes for smear layer removal. After the preparation, the canals were irrigated with 10ml of distilled water and dried with absorbent paper points. Main gutta-percha cones were inserted into the canals and radiograph were taken to verify if they reached the WL and if they fit at the apical third. Fluorescent rhodamine dye was used for mixing the sealers and to properly visualize with confocal laser microscope. The endodontic sealers were inserted into the canals to 1mm short of WL using a 400-rpm lentulo spiral for 5 seconds and with endodontic K-file. Samples were further divided depending on sealers and use of endodontic k file, letulospiral and UA.

[1] Group 1- Endomethasone root canal sealer will be applied with endodontic k-file.

[2] Group 2-Endomethasone root canal sealer will be applied with lentulo spiral.

[3] Group 3- Endomethasone root canal sealer will be applied with lentulo spiral + UA.

[4] Group 4- AH PLUS root canal sealer will be applied with endodontic K-file.

[5] Group 5- AH PLUS root canal sealer will be applied with lentulo spiral.

[6] Group 6- AH PLUS root canal sealer will be applied with lentulo spiral + UA.

[7] Group 7- Sealapex root canal sealer will be applied with endodontic K-file.

[8] Group 8-Sealapex root canal sealer will be applied with lentulo spiral.

[9] Group 9- Sealapex root canal sealer will be applied with lentulo spiral +UA.

[10] Group 10- Bio-C root canal sealer will be applied with endodontic k-file.

[11] Group 11- Bio-C root canal sealer will be applied with lentulo spiral.

[12] Group 12- Bio-C root canal sealer will be applied with lentulo spiral + UA.

A single operator performed all the experimental procedures. UA was performed using non-cutting tip adapted into an ultrasonic device. As the ultrasonic oscillates in a single plane, the tip was activated for 20sec in the buccolingual direction and 20 seconds in the mesiodistal direction of the root canal, 2mm short of the WL. Next, root canal obturation was performed inserting the main gutta-percha cone into the WL followed by lateral compaction technique with a spreader, inserted up to 2mm shorted of the WL and accessory gutta-percha points. Heat plugger removed excess gutta-percha, Cold vertical compaction was done. The cervical portion of the roots were sealed using a temporary filling material. Samples were sectioned using a diamond disc under continuous water cooling to prevent frictional heat, obtaining three slices per sample, one of each root third, with a thickness of 1mm. The samples were washed with distilled water to remove the debris eventually generated during the cut procedures. Slices corresponding to the coronal, middle and apical thirds were analysed in a confocal laser microscope. For correct visualization of all images, the slices were analysed 10 ums below the surface using x10 lens. Respective absorption of rhodamine dye was 545/740 nm. Images were recorded at 10x magnification using the fluorescent mode to a size of 800x800 pixels. Adobe Photoshop was used to measure sealer penetration within dentinal tubules.

## Statistical analysis:

Data was collected and entered into MS EXCEL for statistical analysis. All statistical analysis was done in SPSS version 26. Shapiro-Wilk test was done to verify the normality of data from all analyses. The student's T-test was used to compare the intratubular penetration values of the same sealer within each root third with and without UA. One-way ANOVA and Bonferroni and Kruskal-Wallis and Dunn's post-hoc tests were used to compare all the sealers in each root third regarding intratubular penetration, respectively. Among different sealer bioceramic sealer had maximum SP (p value < 0.05) whereas endomethasone had least SP. Effect of ultrasonic activation on the SP of different sealer was also statistically significant. UA was found to increase SP.

## Results:

Sealer penetration was measured in micrometer by calculating the distance from the canal wall to the maximum level of penetration in the dentinal tubules. Using Adobe Photoshop, the sealer penetration area inside dentinal tubules was calculated. Bio ceramic sealers are the most effective in terms of intratubular penetration as compared to AH PLUS, Sealapex and endomethasone. Sealer penetration was maximum in the coronal region followed by middle and apical region. Sealer penetration in coronal third, middle third and apical third is shown in Table 1, Table 2 and Table 3 respectively.

## Discussion:

The primary cause of pulp deterioration, which in turn causes apical periodontitis, is microorganisms. The use of ultrasonic activation of root canal sealers can possibly favor its penetration inside the dentinal tubules, providing an increased tubular penetration, increased bond strength, less presence of gaps and increased antimicrobial effects. Single-rooted teeth with a single canal were selected for this investigation in order to minimize anatomical variances and achieve uniformity. The crowns were removed at the cemento-enamel junction to produce a standardized length of the root to 16mm of all samples. The root canals were cleaned and shaped using Neoflex files file system. Working length was kept 1 mm short from the apical foramen.

During instrumentation, the canals were flushed with 20ml of 2.5% sodium hypochlorite (NAOCL) irrigation followed by 5ml of ethylenediaminetetraacetic acid (EDTA) 17% during 5 minutes for smear layer removal. After the preparation, the canals were irrigated with 10ml of distilled water and dried with absorbent paper points. Main gutta-percha cones were inserted into the canals and radiograph were taken to verify if they reached the WL and if they fit at the apical third. The sealers were applied using K-file, lentulospiral and lentulospiral + Ultrasonic activation (20s in buccolingual direction and 20s in mesiodistal direction), as per manufacturers instruction. Next, root canal obturation was performed inserting the main gutta-percha cone into the WL followed by lateral compaction technique with a spreader, inserted up to 2mm shorted of the WL and accessory gutta-percha points. A hot plugger was used to remove the surplus gutta-percha 1 mm below the canal opening and cold vertical compaction was carried out. The cervical portion of the roots were sealed using a temporary filling material. Samples were sectioned to obtain 3 slices (one each from coronal 3rd, middle 3rd and apical 3rd) using a diamond disc under continuous running water to obtain uniform slices of 1mm thickness each. Then the samples were observed under SEM to determine intratubular penetration [12]. Studies concluded that ultrasonic activation of endodontic sealer significantly increases push out bond strength [13]. The irrigation solution is directly affected by ultrasonic activation, which also causes debris to be dislodged and turbulence in the solution [14]. When ultrasonic activation is performed a small gap is formed which improves the interfacial adaption, promoting a greater contact area of the sealer with dentinal walls thus a better chemical bonding between sealer and root dentine. Because the coronal root third has more dentinal tubules in terms of quantity and diameter as well as more inter-tubular dentine, which promotes sealer adhesion to dentine walls, lower bond strength was seen in the apical area. Sealer penetration into dentinal tubules could improve sealing of a root filling by increasing the surface contact area between the root filling materials [15]. The chemical elements of sealer cements might have an antibacterial effect that would be strengthened by getting closer to the germs [16]. Scanning electron microscope was used in the study to measure the depth of sealer penetration into dentinal tubules in micrometer. In the present study we found that intratubular sealer penetration was maximum for Bioceramic sealer followed by AH PLUS, Sealapex and endomethasone sealer. The sealer penetration increased significantly following ultrasonic activation for each group. Sealer penetration was more when sealer was applied using lentulospiral as compared to K-file. The results of the sealer penetration in this study were supported by Guimaraes *et al.* (2014) [17], Nikhil *et al.* (2015) [18], Arslan *et al.* (2016) [19], Alcalde *et al.* (2017) [20], Wiesse *et al.* (2018) [21] and Igor Abeu De Benn *et al.* (2020) who reported that ultrasonic activation increases sealer penetration in the dentinal tubules. However, Aksel *et al.* (2019) [22] and Padoin *et al.* (2022) [23], reported that ultrasonic activation does not significantly increases intratubular sealer penetration contradicting the finding of our study. Sealer penetration was more in coronal region followed by middle and least in apical region. This may be the result of inefficient irrigant supply to the apical portion of the canal as well as superior clearance of the smear layer in the coronal and middle levels [24]. The fact that there are fewer tubules at the apical level and that those that are present have smaller diameters or are more often closed could also be contributing factors [25]. A significant component of acoustic streaming is node generation along the activated file, which produces a powerful current along the activated instrument [26].

## Conclusions:

It was concluded that intratubular sealer penetration of various sealers are greatly increased by ultrasonic activation and was maximum in the coronal region followed by middle and apical region.

## Figures and Tables

**Table 1:** Comparison of sealer penetration of different sealers at coronal part in micrometer (Kruskal Wallis test)

**Group 1: Coronal**	**N**	**Endomethasone**	**Sealapex**	**Ah plus**	**Bio c**	**P-value**
		**Mean ± S.D**	**Mean ±S.D**	**Mean ± S.D**	**Mean ±S.D**	
K file	4	101.59 ±3.391	211.17 ± 13.089	225.94 ± 11.958	467.83 ± 19.776	0.001
Lentulospiral	4	108.23 ± 3.944	242.527 ± 10.77	274.29 ± 15.659	512.94 ± 11.211	0.002
Lentulospiral + UA	4	117.815 ± 7.328	282.82 ± 6.661	306.26 ± 18.086	538.20 ± 22.030	0.001

**Table 2:** Comparison of sealer penetration of different sealers at middle part in micrometer (Kruskal Wallis test)

**Group 2: Middle**	**N**	**Endomethasone**	**Sealapex**	**Ah plus**	**Bio c**	**P-value**
		**Mean ± S.D**	**Mean ± S.D**	**Mean ± S.D**	**Mean ± S.D**	
K file	4	84.06 ± 9.77	183.36 ± 7.73	195.03 ± 9.372	282.15 ± 5.688	0.009
Lentulospiral	4	94.63 ± 4.515	201.93 ± 7.622	246 ± 13.776	296.34±5.045	0.004
Lentulospiral + UA	4	94.577 ± 6.932	249. 13 ± 7.88	271.782 ± 16.365	405.59 ± 64.661	0.001

**Table 3:** Comparison of sealer penetration of different sealers at apical part in micrometer (Kruskal Wallis test)

**Group 3: Apical**	**N**	**Endomethasone**	**Sealapex**	**Ah plus**	**Bio c**	**P-value**
		**Mean ± S.D**	**Mean ± S.D**	**Mean ± S.D**	**Mean ± S.D**	
K file	4	64.747 ± 5.87	141.37 ± 15.66	174.25 ± 7.740	247.99 ±6.173	0.001
Lentulospiral	4	73.177 ± 11.445	180.80 ± 9.682	213.07 ± 10.668	265.83 ± 13.806	0.001
Lentulospiral + UA	4	73.81 ± 2.739	219. 33 ± 9.055	240.11 ± 8.387	292.26 ± 5.974	0.002
